# Diversity and Universality of Endosymbiotic *Rickettsia* in the Fish Parasite *Ichthyophthirius multifiliis*

**DOI:** 10.3389/fmicb.2017.00189

**Published:** 2017-02-09

**Authors:** Kassandra E. Zaila, Thomas G. Doak, Hannah Ellerbrock, Che-Huang Tung, Mauricio L. Martins, Daniel Kolbin, Meng-Chao Yao, Donna M. Cassidy-Hanley, Theodore G. Clark, Wei-Jen Chang

**Affiliations:** ^1^Department of Biology, Hamilton College, ClintonNY, USA; ^2^Department of Biology, Indiana University, BloomingtonIN, USA; ^3^National Center for Genome Analysis Support, Indiana University, BloomingtonIN, USA; ^4^Department of Aquatic Biosciences, National Chyai UniversityChyai City, Taiwan; ^5^Departamento de Aquicultura, Centro de Ciências Agrárias, Universidade Federal de Santa CatarinaFlorianópolis, Brazil; ^6^Department of Microbiology and Immunology, College of Veterinary Medicine, Cornell University, IthacaNY, USA; ^7^Institute of Molecular Biology, Academia SinicaTaipei, Taiwan

**Keywords:** Ciliophora, alphaproteobacteria, Sphingobacteria, hyperparasitism, phagocytosis, symbiosis

## Abstract

Although the presence of endosymbiotic rickettsial bacteria, specifically *Candidatus* Megaira, has been reported in diverse habitats and a wide range of eukaryotic hosts, it remains unclear how broadly *Ca.* Megaira are distributed in a single host species. In this study we seek to address whether *Ca.* Megaira are present in most, if not all isolates, of the parasitic ciliate *Ichthyophthirius multifiliis*. Conserved regions of bacterial 16S rRNA genes were either PCR amplified, or assembled from deep sequencing data, from 18 isolates/populations of *I. multifiliis* sampled worldwide (Brazil, Taiwan, and USA). We found that rickettsial rRNA sequences belonging to three out of four *Ca.* Megaira subclades could be consistently detected in all *I. multifiliis* samples. *I. multifiliis* collected from local fish farms tend to be inhabited by the same subclade of *Ca.* Megaira, whereas those derived from pet fish are often inhabited by more than one subclade of *Ca.* Megaira. Distributions of *Ca.* Megaira in *I. multifiliis* thus better reflect the travel history, but not the phylogeny, of *I. multifiliis*. In summary, our results suggest that *I. multifiliis* may be dependent on this endosymbiotic relationship, and the association between *Ca.* Megaira and *I. multifiliis* is more diverse than previously thought.

## Introduction

Rickettsial bacteria (Order Rickettsiales), members of alphaproteobacteria, are well-known as the causative agents for insect-borne human diseases such as typhus, scrub typhus, and Rocky Mountain spotted fever (Walker and Ismail, 2008). These bacteria are gram-negative, obligate intracellular organisms, and their presence was once thought to be limited to animals, particularly insects and vertebrates (Raoult and Roux, 1997). Recently, surveys of environmental samples revealed that in addition to the pathogenic rickettsia, rickettsia-like bacteria could be found as endosymbionts in a variety of species and from different habitats. However, the functions of these rickettsial endosymbionts in their hosts remain unclear.

Results derived from phylogenetic analyses using 16S rRNA sequences show that rickettsia-like endosymbiotic bacteria can be classified into two monophyletic groups. The first group, the recently described *Candidatus* Midichloriaceae (Vannini et al., 2005; Epis et al., 2008; Gillespie et al., 2012; Mariconti et al., 2012; Williams-Newkirk et al., 2012; Driscoll et al., 2013; Montagna et al., 2013), is placed as a sister clade to Anaplasmataceae, and comprises endosymbionts found in insects (Epis et al., 2008; Hornok et al., 2008; Erickson et al., 2009; Richard et al., 2009; Matsuura et al., 2012), amoebas (Fritsche et al., 1999), ciliates (Vannini et al., 2010; Boscaro et al., 2013a,b), placozoa (Driscoll et al., 2013), and cnidarians (Fraune and Bosch, 2007; Sunagawa et al., 2009). Furthermore, members of *Midichloriaceae* have also been detected in fish suffering from strawberry disease (Lloyd et al., 2008, 2011) and red mark syndrome (Metselaar et al., 2010; Cafiso et al., 2016), and in humans and other mammals after tick bites (Mediannikov et al., 2004; Mariconti et al., 2012; Matsuura et al., 2012; Bazzocchi et al., 2013). However, there has been no direct evidence suggesting that these *Midichloriaceae* are etiological agents of disease.

The other group of rickettsia-like endosymbiotic bacteria, *Candidatus* Megaira, forms a sister clade to the genus *Rickettsia* (family Rickettsiaceae) (Schrallhammer et al., 2013). Based on SSU rRNA sequences, Schrallhammer et al. (2013) further classified *Ca.* Megaira into three subclades. Members of the subclade *Ca.* Megaira polyxenophila were identified in both marine and freshwater ciliates (Vannini et al., 2005), in green algae (Kawafune et al., 2012), in lake water from the US (Percent et al., 2008) and China, in subsurface water from South Africa, and in aquaria in Greece (Vlahos et al., 2013). The other two subclades, *Ca.* Megaira B and C, contain species found in diverse hosts and habitats including: ciliate *Ichthyophthirius multifiliis* (Sun et al., 2009), cnidarians (Fraune and Bosch, 2007; Sunagawa et al., 2009), siphonous green algae (Hollants et al., 2013), lake water from the US (Percent et al., 2008), water from a lagoon in North Pacific (Galand et al., 2012), and a wastewater treatment plant in France (Chouari et al., 2010). There have been no reports that these bacteria are pathogenic, and the growth and reproduction of ciliate *Diophrys* were not affected when inhabited by *Ca.* Megaira (Vannini et al., 2003).

While it seems that *Ca.* Megaira are widely spread, it is not clear how ubiquitous they are. Furthermore, how universal these bacteria are in isolates/populations of particular host species is less well-studied. Research carried out by Kawafune et al. (2012) showed that *Ca.* Megaira were present only in 1 of 12 isolates of four unicellular green algal species (*Cateria*), and in one of nine isolates of multicellular green algae *Volvox carteri* (Kawafune et al., 2014), suggesting that *Ca.* Megaira might not be ubiquitously found in all isolates of one species. However, despite the works on non-phagotrophic green alga, to our knowledge there have been no other research systematically examining the distribution of *Ca.* Megaira in one single species, particularly in phagotrophic ones.

The parasitic ciliate *I. multifiliis* is the etiological agent for the ‘white spot disease’ in freshwater fish (Matthews, 2005; Dickerson, 2011). *I. multifiliis* contains an oral apparatus (Dickerson, 2006), and are apparently phagotrophic (Lobo-da-Dunha and Azevedo, 1993). Moreover, endosymbiotic Sphingobacteria and rickettsial alphaproteobacteria were detected in two *I. multifiliis* isolates isolated from the state of Georgia, USA (Sun et al., 2009; Coyne et al., 2011). The rickettsial alphaproteobacteria were later identified as members of the *Ca.* Megaira subclade C (Schrallhammer et al., 2013). We are therefore intrigued to determine if *Ca.* Megaira can be detected in most, if not all, isolates of the phagotrophic *I. multifiliis*. Furthermore, the phylogenetic relationships among different isolates of *I. multifiliis* can now be well-resolved by using mitochondrial sequences (MacColl et al., 2015). The phylogenies of *Ca.* Megaira, if they are present in most isolates of *I. multifiliis*, can then be compared to that of *I. multifiliis* to help deduce transmission routes of *Ca.* Megaira.

In this study we show that *Ca.* Megaira can be detected in 18 isolates of *I. multifiliis*, collected from Brazil, Taiwan, and the US. *Ca.* Megaira can now be classified into four subclades based on their rRNA sequences, and at least three of the subclades are capable of inhabiting *I. multifiliis*. The significance of the ubiquitous distribution of *Ca.* Megaira in *I. multifiliis*, and the transmission routes of *Ca.* Megaira, are discussed.

## Materials and Methods

### *I. multifiliis* and DNA Isolation

*Ichthyophthirius multifiliis* was collected from infected fish in the US, Taiwan, and Brazil, and each isolate likely derived from a distinct population. This collection represents more than 20 years of effort—on many researchers’ part—in the collection and storage of samples from fish farms and pet stores across the world (**Table 1**). Isolates were named with a letter(s) denoting the state or the country of its origin and a sequential number in the order they were discovered (**Table 1**). Among the 18 *I. multifiliis* isolates 9 have been previously reported (Lin et al., 1996; MacColl et al., 2015), but only endosymbionts in the G5 isolate have been studied (Sun et al., 2009). Isolates collected in the US were at one point in time cultivated in the lab following previously established protocols (Noe and Dickerson, 1995), and except for G15 and NY3, all other US isolates were clonal lines. *I. multifiliis* trophont cells were collected from infected fish either by gently rubbing the skin of fish (Cassidy-Hanley et al., 2011), or by using saline shock (Schmahl et al., 1989). DNA was extracted either following protocols described elsewhere (Cassidy-Hanley et al., 2011; MacColl et al., 2015), or using the Qiagen DNeasy Blood & Tissue Kit (Redwood City, CA, USA) following manufacturer’s protocols. The protocol of using fish (to pass *I. multifiliis*) was approved by the Institutional Animal Care and Use Committee of Cornell University (protocol number 1996-0083).

**Table 1:** Histories and characteristics of the 17 *Ichthyophthirius multifiliis* isolates used in this study.

Isolate name	Location of isolation	Date	Host	Parasite stage
Ark1	Keo Fish Farm, Keo, AR, USA	2004	Hybrid Stripped Bass	Theront
Ark2	U. of Arkansas at Pine Bluff (Hatchery)	2005	Channel catfish	Theront
Ark5	Central Arkansas	2005	Channel catfish	Theront
Ark7	Stoneville, MS, USA	2008	Channel catfish	Tomont
Ark9	Lonoke, AR, USA	2008	Golden shiner	Tomont
Ark10	Stuttgart, AR, USA	2011	Blue catfish	Theront
Ark11	Lonoke, AR, USA	2013	Channel catfish	Theront
Ark12	Hot Springs State Hatchery, Hot Springs, AR, USA	2014	White bass	Theront
BR1	Paulo Lopes municipality, Brazil	2014	Silver catfish	Trophont
G15	Supermarket, Athens, GA, USA	2011	Red parrot fish	Tomont
NY3	Petstore, Ithaca, NY, USA	2004	Oscar	Theront
NY4	Petstore, Ithaca, NY, USA	2004	Freshwater shark	Theront
NY6	Ithaca, NY, USA	2005	Goldfish	Theront
NY7	Supermarket, New Hartford, NY, USA	2010	Oscar	Theront
TW1	Chianan Irrigation system, Chyayi, Taiwan	2014	Rosy bitterling	Trophont
TW5	Chyayi, Taiwan (Tailand, imported)	2015	Rainbow fish	Trophont
TW7	Chyayi, Taiwan (Tailand, imported)	2015	Kuhli loach	Trophont


### Amplification, Cloning, and Sequencing

Endosymbiotic bacterial 16S rRNA sequences were either PCR amplified, or derived from whole genome assemblies. PCR mixtures contained 1X GoTaq Green Master Mix (Promega, Madison, WI, USA), each primer at 0.2 μM, and DNA (5–50 ng) in a final volume of 50 μL. A reagent negative control was always included in every PCR experiment. PCR primers were either the bacterial SSU-specific set described elsewhere (Weisburg et al., 1991) (*Escherichia coli* rRNA positions 8–1,509, GenBank: J01859.1; Forward 5′ AGA GTT TGA TYM TGG CTC AG 3′, Reverse 5′ GGH TAC CTT GTT ACG ACT 3′), or an in-house set more specific against Rickettsia 16S rRNA (approximate *E. coli* rRNA positions 45–1,345; Forward 5′ TGC TTA ACA CAT GCA AGT CGA ACG A 3′, Reverse 5′ TAG TGA TTC CGA CTT CAT GCT CT 3′). The following cycling conditions were followed: initial denaturation at 94°C for 2 min, denaturation at 94°C for 30 s, annealing at 46°C for 30 s, extension at 72°C for 1.5 min (30 cycles), with a final extension of 68°C for 5 min. Amplified PCR products were cloned into pGEM-T Easy Vector (Promega), and sequences were determined by using Sanger’s sequencing method (Genewiz, South Plainfield, NJ, USA). For each isolate at least 10 positive clones were screened. rRNA sequences are deposited in NCBI GenBank (accession KT851755-851878).

We followed protocols described elsewhere to amplify and determine *I. multifiliis* mitochondrial *cox-1* and *nad1_b* sequences (MacColl et al., 2015). Briefly, 0.2 μM of each primer (*cox-1* Forward: 5′ TATCAGGTGCTGCATTAGCTACT 3′, Reverse: 5′ TAAACCTAAAGTAGATGAAGTGTGAAG 3′; *nad1_b* Forward: 5′ CTATGACCATAAATCGGAGAAAGTT 3′, Reverse: 5′ GAGTTTATATCATGGAAGCTAACAG 3′), and 2–20 ng of *I. multifiliis* DNA were added to a PCR mixture containing 1X GoTaq in a final volume of 50 μL. Cycling conditions were: 95°C 2 min followed by 35 cycles of 95°C 30 s, 50°C 1 min, 72°C 1.5 min, with a final extension of 72°C for 5 min. *Cox-1* and *nad1_b* sequences are also deposited in NCBI GenBank (KT783590–KT783607).

Whole genomes of isolates G15, Ark11, and Ark12 were sequenced using Illumina technologies, which generated paired reads with >200X coverages of *I. multifiliis* genome (MacColl et al., 2015). Raw reads were first corrected using SOAPec v2.01 (Luo et al., 2012), and corrected reads specific to bacterial 16S rRNA were baited using MIRA v4.9.3 (Chevreux et al., 2004) against the bacterial 16S rRNA database v119 downloaded from The SILVA ribosomal RNA database (Quast et al., 2013; Yilmaz et al., 2014) before being assembled by MIRA into contigs.

### Phylogenetic Analyses

DNA sequences were first aligned using T-Coffee (Notredame et al., 2000), and alignments were further manually corrected in Jalview (Waterhouse et al., 2009) and/or BioEdit (Hall, 1999). For phylogenetic tree reconstructions protocols described elsewhere were followed (MacColl et al., 2015). Briefly, maximum likelihood (ML) trees were constructed with models predetermined by jModeltest (rRNA sequences: GTR+G; concatenated *cox-1* and *nad1_b*: TIM1+I) (Darriba et al., 2012) and 1,000 bootstrapping replicates (Guindon and Gascuel, 2003). For Bayesian analyses (MB), MrBayes was used with the setting of: GTR+G model, two independent runs (each with three heated chains and one cold chain), 2,500,000 MCMC steps, and a sampling frequency of 1,000 (Ronquist et al., 2012). By the end of MCMC the standard deviation of split frequencies reached 0.0068. A burn-in of 25%, or 625, was used to generate both parameters and the consensus tree.

## Results

### Phylogeny of *Ca.* Megaira

We identified more than 50 unique rickettsia-like 16S rRNA sequences from 17 isolates of *I. multifiliis*, and at least one sequence was detected in each of the isolates. The sequence of the rickettsial endosymbiotic bacterium found in the 18th isolate, G5, was published in an earlier report (Sun et al., 2009). If there were no deviations between two sequences beyond three or more positions, or 0.23% difference among 1,302 positions including gaps, only one was chosen for subsequent analyses. After such filtration 42 sequences were retained.

**FIGURE 1:**
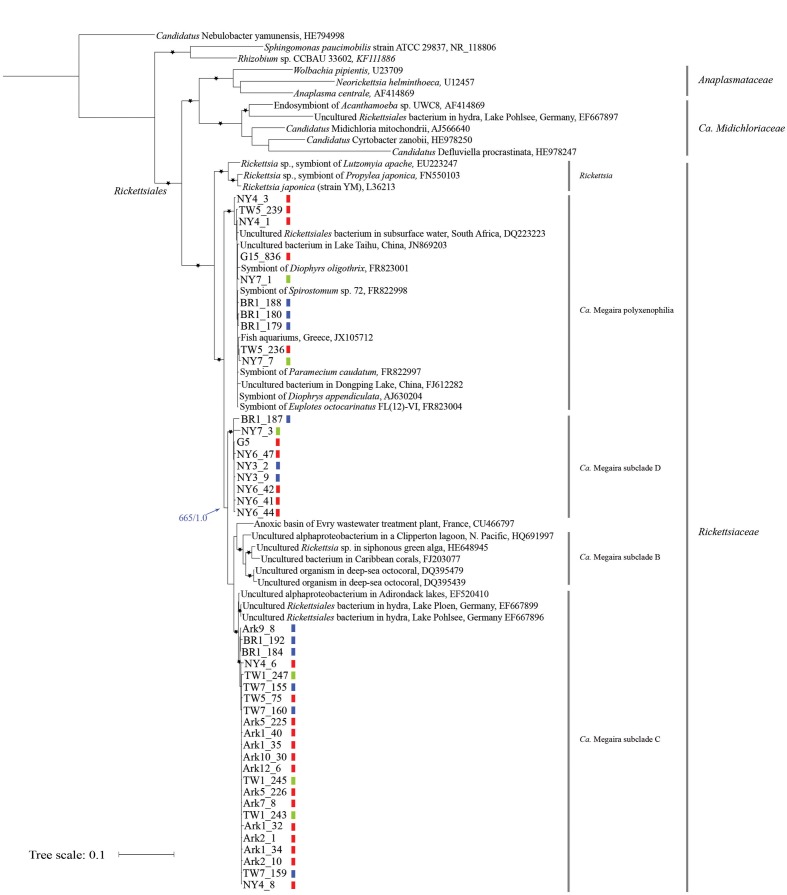
**Maximum likelihood (ML) tree derived from 16S rRNA sequences of *Ca*.** Megaira and representative Alphaproteobacteria. Asterisks (^∗^) denote branches with >750 bootstrap values and >0.75 posterior probability in Bayesian (MB) analyses. The blue arrow and numbers indicate bootstrap support from ML and posterior probability from MB analyses on the branch separating *Ca*. Megaira subclades B, C, and D from subclade polyxenophila. The genus and family names in the order Rickettisales are provided on the right. Short vertical color bars indicate the three phylogroups of *Ichthyophthirius multifiliis* (see **Figure 2**). Ark11 share identical *Ca*. Megaira 16S rRNA to that of Ark12_6 and is not shown. Scale bar represents 0.1 nucleotide substitutions per site.

Phylogenetic analyses were conducted to assess the relationships among the newly obtained sequences and *Ca.* Megaira 16S rRNA sequences reported in other studies, including those derived from endosymbiotic bacteria found in ciliates (Vannini et al., 2005; Schrallhammer et al., 2013), hydra (Fraune and Bosch, 2007), corals (Sunagawa et al., 2009), siphonous green algae (Hollants et al., 2013), and from environmental samples (lakes (Percent et al., 2008), stratified lagoon (Galand et al., 2012), basins of a wastewater treatment plant (Chouari et al., 2010), aquariums (Vlahos et al., 2013). In addition, sequences derived from representative species in the families of Rickettsiaceae, Midichloriacea, and Anaplasmataceae, from two non-Rickettsiales alphaproteobacteria were included as internal reference points. *Candidatus* Nebulobacter yamunensis, an endosymbiotic gammaproteobacteria found in the ciliate *Euplotes aediculatus* (Boscaro et al., 2012), was used as the outgroup (**Figure 1**).

Both Bayesian (MB) and ML trees place all newly reported rickettsia-like 16S rRNA sequences and *Ca.* Megaira sequences in a monophyletic group next to the genus *Rickettsia*, a result consistent with a previous observation (Schrallhammer et al., 2013) (**Figure 1**). From this point we will follow the nomenclature system established by Schrallhammer et al. (2013) and collectively regard all endosymbiotic bacteria in this monophyletic group as *Ca.* Megaira.

Based on our phylogenetic analyses, *Ca.* Megaira can be further divided into four well-supported subclades, and at least three of the subclades are capable of inhabiting *I. multifiliis* (**Figure 1**). The early diverging position and the grouping of the subclade *Ca.* Megaira polyxenophilia are consistent with previous findings (Schrallhammer et al., 2013). However, *Ca.* Megaira polyxenophilia was not known to inhabit *I. multifiliis* and here we show ample examples (**Figure 1**).

The remaining three subclades might have descended from a common ancestor after the split from *Ca.* Megaira polyxenophilia (blue arrow in **Figure 1**), but the bootstrapping support of the branching point from ML analyses is only moderate. Among these three subclades, the grouping of the subclade *Ca.* Megaira B is consistent with findings reported in other studies (Schrallhammer et al., 2013), and this subclade has been the only one that has not been detected in *I. multifiliis. Ca.* Megaira subclade C includes endosymbionts found in hydra, in a US lake sample, and in *I. multifiliis* (**Figure 1**). It should be noted, however, that while the rickettsial endosymbionts in the G5 isolate of *I. multifiliis* were first classified as members in *Ca.* Megaira subclade C (Schrallhammer et al., 2013), our results show that these and a few other rickettsial endosymbionts detected in other *I. multifiliis* isolates should be classified as a new *Ca.* Megaira subclade D (**Figure 1**). Moreover, to date rickettsial endosymbionts in *Ca.* Megaira subclade D have only been detected in *I. multifiliis*. A nucleotide blast search using the *Ca.* Megaira 16S rRNA sequence found in G5 against the NCBI nr/nt database failed to identify sequences—including those derived from environmental samples—with higher similarities than those found in *Ca.* Megaira subclade C (data not shown).

### Phylogeny of *I. multifiliis*

**FIGURE 2:**
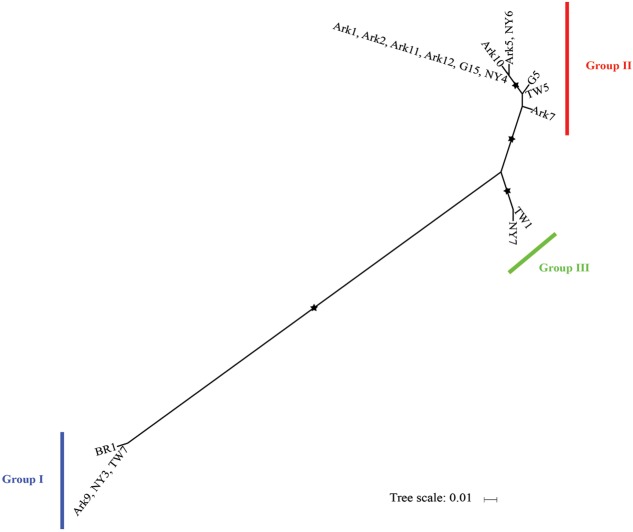
**Maximum likelihood tree derived from concatenated mitochondrial *cox-I* and *nad1_b* sequences of 18 *I. mutifiliis* isolates.** Asterisks (^∗^) denote branches with >750 bootstrap values. Color bars indicate the three distinct phylogroups. Scale bar represents 0.01 nucleotide substitutions per site.

We then determined the phylogeny of the 18 host isolates of *I. multifiliis* using concatenated mitochondrial *cox-1* and *nad1_b* sequences. In a previous study based on nine isolates *I. multifiliis* could be classified into two distinct groups with the possibility of a third group (MacColl et al., 2015). With more isolates included in this study it is clear that the 18 isolates of *I. multifiliis* belong to three distinct groups (**Figure 2**).

Group II contains seven out of eight isolates collected from Arkansas, demonstrating a pattern of local, repetitive infection of fish stocks. This pattern matches the isolate histories: most of these isolates were collected from local hatchery farms (**Table 1**). In contrast, for isolates obtained from pet stores (**Table 1**) where the sources of *I. multifiliis* are expected to be variable, such a pattern does not exist. For instance, among the four isolates obtained in New York State, NY3, NY4, and NY7 were obtained from three different pet stores and belong to Groups I, II, and III, respectively (**Figure 2**). A similar pattern holds for isolates TW5 (Group II) and TW7 (Group I), which were imported from vendors in Thailand to aquarium shops in Taiwan (**Figure 2**).

### Distributions of *Ca.* Megaira in *I. multifiliis*

We next mapped groupings of *I. multifiliis* to the phylogenetic tree of *Ca.* Megaira (**Figure 1**, short vertical color bars). While in most *I. multifiliis* isolates we could only detect *Ca.* Megaira endosymbionts from one subclade, a few isolates, particularly those derived from pet store aquaria—NY4, NY7, and TW5—harbored endosymbionts from two subclades. BR1 was inhabited with endosymbionts from three *Ca.* Megaira subclades. The detailed history of this isolate is, however, not clear.

### Transmissions of *Ca.* Megaira

If *Ca.* Megaira are transmitted solely vertically, i.e., to sister cells through asexual division, we should expect congruent evolution between *Ca.* Megaira and *I. multifiliis*. The distributions of *Ca.* Megaira in *I. multifiliis* isolates show that a congruent pattern does not exist (**Figure 1**). The three *Ca.* Megaira subclades that are found capable of inhabiting *I. multifiliis* can be detected in all three groups of *I. multifiliis*. This observation would argue strongly against solely vertical transmission.

Furthermore, we found that all isolated samples from Arkansas, which fell into two distinct groups, were inhabited with *Ca.* Megaira subclade C, suggesting a strong influence of the local environment on the acquisition of endosymbiont strains.

### The Presence of Sphingobacteria in *I. multifiliis*

Since Sphingobacteria were also detected in two isolates of *I. multifiliis* collected from Georgia, USA (Sun et al., 2009), we set out to determine whether Sphingobacteria, like *Ca.* Megaira, were present in all 18 isolates of *I. multifiliis*. In three isolates, G15, Ark11, and Ark12, where genomic sequences were determined using next generation sequencing technologies with high coverages, assembled contigs with high similarity (>90%) to Sphingobacteria 16S rRNA sequence (GQ870456.1) were not detected (data not shown). We also failed to detect Sphingobacteria or Sphingobacteria-like 16S rRNA sequences in cloned PCR products. These results suggest that Sphingobacteria is not present in all *I. multifiliis* isolates.

## Discussion

In this study we surveyed the presence and distribution of rickettsial endosymbionts *Ca.* Megaira in 18 isolates of the parasitic ciliate *I. multifiliis*. In contrast to findings reported in non-phagotrophic green alga, where only a few isolates harbored this endosymbiont (Kawafune et al., 2012, 2014), *Ca.* Megaira could be detected in all 18 isolates of *I. multifiliis*, collected from North and South America, and Southeast Asia. Like many other ciliates, *I. multifiliis* has an oral apparatus and is presumed to be phagocytic when feeding on fish (Ewing et al., 1985; Dickerson, 2006). It is therefore possible that *I. multifiliis* acquires *Ca.* Megaira through phagocytosis and *Ca.* Megaira subsequently escapes from phagolysosomes using a mechanism similar to that used by pathogenic rickettsiae (Teysseire et al., 1995; Whitworth et al., 2005). On the other hand, ciliates are also equipped with a clathrin-mediated endocytosis pathway (Ramoino et al., 2001; Elde et al., 2005). Because rickettsiae invade mammalian cells in a clathrin-dependent manner (Chan et al., 2009), it is possible that *Ca.* Megaira enters *I. multifiliis* through clathrin-mediated endocytosis. Further experiments are needed to elucidate which mechanism(s) *Ca.* Megaira use to enter their hosts.

The ubiquitous presence of *Ca.* Megaira in *I. multifiliis* prompts us to consider whether the bacteria and the ciliate host have formed a dependent relationship, which may well be an example of hyperparasitism between bacteria (hyperparasite) and protozoan (pathogen) (Parratt and Laine, 2016). *I. multifiliis* shows a significant reduction of its somatic genome size (∼50 Mb) compared to two other free-living ciliate species in the same class (Oligohymenophorea) – *Paramecium tetraurelia* (72 Mb) (Aury et al., 2006) and *Tetrahymena thermophila* (104 Mb) (Eisen et al., 2006). Although parasitic protozoans tend to have smaller genomes (Ersfeld, 2003; Hertz-Fowler et al., 2005), *I. multifiliis* might have undergone further genome reduction due to the formation of a mutualistic symbiotic relationships with *Ca.* Megaira.

Prokaryotic endosymbionts have been identified in more than 200 ciliate species (Fokin, 2004; Schweikert et al., 2013), and these endosymbionts have been shown to provide hosts with nutritional support (Vannini et al., 2003), defense (Beale et al., 1969; Preer et al., 1972; Quackenbush and Burbach, 1983), and/or access to better environments (Finlay and Fenchel, 1989; Fenchel and Finlay, 1991a,b). It is unclear what roles *Ca.* Megaira may play in *I. multifiliis* biology. Lobo-da-Cunha and Azevedo showed that endosymbiotic bacteria in *I. multifiliis*, likely *Ca.* Megaira, were surrounded by glycogen in the cytoplasm (Lobo-da-Cunha and Azevedo, 1988). While this raises the interesting possibility that *Ca.* Megaira utilizes glycogen/glucoses derived from *I. multifiliis*, it does not immediately suggest that *Ca.* Megaira provides anything to its *I. multifiliis* host. Further details on metabolic dependencies between *Ca.* Megaira and *I. multifiliis* may be revealed when genomic sequences of *Ca.* Megaira become available.

With the addition of *Ca.* Megaira 16S rRNA sequences derived from *I. multifiliis* it is now clear that *Ca.* Megaira can be further divided into at least four subclades. Three of these subclades (polyxenophila, C and D) are capable of inhabiting *I. multifiliis*, while subclade B, which is found primarily in seawater samples, is not (**Figure 1**). The geographic isolation and/or changes in host tropism may contribute to this phenomenon (*I. multifiliis* only infect freshwater fish). Along the same lines, the fact that *Ca.* Megaira subclade D appears to be present only in *I. multifiliis* suggests a specific tropism for these bacteria, although under-sampling may explain this as well.

By comparing the phylogenies of *I. multifiliis* and *Ca.* Megaira, we concluded that transmission of *Ca.* Megaira is not solely vertical. Isolates of *I. multifiliis* in the same phylogroup may be inhabited with different subclades of *Ca.* Megaira. Moreover, local *Ca.* Megaira strains seem to play a more important role in determining which bacteria are present in *I. multifiliis*. The fact that all Arkansas isolates of *I. multifiliis*, regardless their phylogroups, were inhabited with *Ca.* Megaira subclade C, and not with other subclades, supports this idea. In this case, local bacteria may transmit horizontally to *I. multifiliis* and outcompete *Ca.* Megaira already inhabiting *I. multifiliis*. The presence of two subclades of *Ca.* Megaira in some clonally derived parasite lines (for example, NY4 and NY7), may reflect complex travel histories (exposures to different *Ca.* Megaira in different locations) and balanced competition between endosymbiont groups. The host/bacterial nature histories may always be more complicated than simple scenario we propose. Multiple gains/losses, in combination with horizontal/vertical transmissions, could result in what we observe today. More molecular sequences from *Ca.* Megaira will allow us to make better phylogenetic inferences not only between *Ca.* Megaira subclades, but also within subclades, with which we will be better able to determine the transmission routes of *Ca.* Megaira.

Finally, host range and tropisms of *Ca.* Megaira remain to be further investigated. *Ca.* Megaira polyxenophilia is capable of inhabiting at least six ciliate species encompassing three distinct classes: Hypotrichea (*Diophrys oligothrix. Diophrys appendiculata*, and *Euplotes octocarinatus*), Heterotrichea (*Spirostomum* sp.), and Oligohymenophorea (*Paramecium caudatum* and *I. multifiliis*). Do these observations imply that *Ca.* Megaira (polyxenophilia) may be able to inhabit most, if not all, species in these three classes? Moreover, could *Ca.* Megaira subclade C and subclade D inhabit ciliate species other than *I. multifiliis*? In pathogenic rickettsiae it has been shown that several surface proteins, e.g., rOmpB (Sca5), Sca1, and Sca2, play significant roles in host cell adherence and invasion, and may be involved in determining host tropisms (Cardwell and Martinez, 2009; Chan et al., 2009; Riley et al., 2010; Uchiyama, 2012). An extensive survey of variations of these protein sequences from different *Ca.* Megaira subclades may help us gain insight on the host specificities of *Ca.* Megaira.

## Ethics Statement

The protocol of using fish (to pass *I. multifiliis*) was approved by the Institutional Animal Care and Use Committee of Cornell University (protocol number 1996-0083).

## Author Contributions

KZ, TD, HE, C-HT, M-CY, DC-H, TC, and W-JC conceived and designed the study. KZ, HE, C-HT, MM, and DK conducted experiments. KZ, TD, C-HT, TC, and W-JC analyzed the resulting data. KZ, TD, and W-JC wrote the manuscript. KZ, TD, M-CY, DC-H, TC, and W-JC revised the manuscript.

## Conflict of Interest Statement

The authors declare that the research was conducted in the absence of any commercial or financial relationships that could be construed as a potential conflict of interest.
